# Changes in Clinical Manifestations and Course of Systemic Lupus Erythematosus and Secondary Antiphospholipid Syndrome over Three Decades

**DOI:** 10.3390/biomedicines11041218

**Published:** 2023-04-19

**Authors:** Nikolett Nagy, Gábor Papp, Eszter Gáspár-Kiss, Ágnes Diószegi, Tünde Tarr

**Affiliations:** 1 Division of Clinical Immunology, Institute of Internal Medicine, Faculty of Medicine, University of Debrecen, H-4032 Debrecen, Hungarypapp.gabor@med.unideb.hu (G.P.);; 2 Division of Metabolic Disorders, Institute of Internal Medicine, Faculty of Medicine, University of Debrecen, H-4032 Debrecen, Hungary

**Keywords:** systemic lupus erythematosus, antiphospholipid syndrome, disease course, therapy

## Abstract

Systemic lupus erythematosus (SLE) is often associated with antiphospholipid syndrome (APS), which potentially results in a more severe disease course and reduced life expectancy. Since the therapeutic guidelines have been refined in the last 15 years, we assumed that the diseases course has become more favorable. In order to shed light on these achievements, we compared the data of SLE patients diagnosed before and since 2004. In our retrospective study, we assessed a wide spectrum of clinical and laboratory data of 554 SLE patients who received regular follow-up care and therapy at our autoimmune center. Among these patients, 247 had antiphospholipid antibodies (APAs) without clinical signs of APS, and 113 had definitive APS. In the APS group, among patients diagnosed since 2004, deep vein thrombosis (*p* = 0.049) and lupus anticoagulant positivity (*p* = 0.045) were more frequent, while acute myocardial infarction was less frequent (*p* = 0.021) compared with patients diagnosed before 2004. Among the APA positive patients without definitive APS, anti-cardiolipin antibody positivity (*p* = 0.024) and development of chronic renal failure (*p* = 0.005) decreased in patients diagnosed since 2004. Our study demonstrates that the disease course has changed in recent years; however, in the presence of APS, we have to expect repeated thrombotic events despite adequate anticoagulant therapy.

## 1. Introduction

Antiphospholipid syndrome (APS) is an autoimmune, thrombo-inflammatory disease characterized by the production of circulating antiphospholipid antibodies (APAs), which play central role in the blood clot formation in arteries, veins and small vessels. APAs are a heterogeneous group of immunoglobulins, including lupus anticoagulant (LA), anti-cardiolipin antibodies (aCL), and anti-b2-glycoprotein-I antibodies (ab2GPI), directed against phospholipids, cofactors or phospholipid–cofactor complexes. The detection of their presence is important in the diagnosis of the syndrome; nevertheless, the presence of APAs without any relevant clinical symptoms does not establish an APS diagnosis. Deep veins, pulmonary vessels, and arteries are the most common sites of thrombosis in APS. Furthermore, pregnancy morbidities such as miscarriage, intrauterine fetal death, preeclampsia and eclampsia resulting in premature birth are also common clinical manifestations. The disease can occur as primary APS, but when associated with a systemic autoimmune disease, most commonly SLE, the syndrome is defined as secondary APS [1,2,3].

Systemic lupus erythematosus (SLE) is a chronic systemic autoimmune disease potentially affecting various tissues and organ systems such as cutaneous, renal, cardiopulmonary, musculoskeletal, neural, and hematologic systems. The course of the disease usually consists of alternating periods of remission and exacerbation of mild to moderate severity, which can lead to serious long-term consequences [4,5]. The relationship between the development of APS and SLE is quite close. Based on the Euro Phospholipid Project’s data, 36% of APS patients suffer from SLE as well [6]. According to other reports, 20–40% of SLE patients carry antiphospholipid antibodies, out of whom 50–70% will evolve into patients with definitive APS within 20 years [7]. The connection between the two diseases is also shown by the fact that patients diagnosed with primary APS may later develop SLE [8]. Both disease spectrums have several overlapping clinical manifestations, such as hemolytic anemia, thrombocytopenia, leuko-lymphopenia, neurological symptoms, renal impairment or livedo reticularis [9]. It was demonstrated that the development of secondary APS in lupus patients worsens chronic organ damage and increases mortality [10]. The incidence of cardiovascular morbidities and thrombosis is increased in SLE patients compared with the general population, and the accelerated atherosclerosis may be explained by lupus associated risk factors in addition to traditional risk factors. The presence of APAs further increases the chance of vascular diseases. Deep vein thrombosis, pulmonary embolism, pregnancy morbidities, heart valve disorders, pulmonary hypertension, thrombocytopenia, hemolytic anemia, nephropathy and cognitive dysfunction are more prevalent in APA positive lupus patients than in APA negative ones [11]. Therefore, thrombosis prophylaxis is particularly important in SLE patients with associated APS. Nevertheless, the results of studies on primary prophylaxis in APA positive SLE patients are controversial; furthermore, studies on immunmodulant and immunosuppresive treatments in APS patients are limited only to case reports and case series [12,13]. Our workgroup has previously investigated the relationship between SLE and APS [14]; however, new therapeutic recommendations have been introduced in the recent decades, which could improve the course of disease and quality of life.

In order to shed light on the achievements of the last decades, we compared the wide spectrum of laboratory and clinical data of SLE patients diagnosed before and since 2004 in order to determine any changes in the disease course.

## 2. Materials and Methods

### 2.1. Study Population

In our retrospective study, we assessed a wide spectrum of clinical and laboratory data of 554 SLE patients who received regular follow-up care and therapy at the Division of Clinical Immunology, Institute of Internal Medicine, Faculty of Medicine, University of Debrecen. The diagnosis of SLE was established on the American College of Rheumatology (ACR) classification criteria (1997) or SLICC (2012) criteria, according to the date of diagnosis [15,16]. Patients diagnosed with SLE before 2012 were revised according to the SLICC criteria for SLE; additionally, all SLE patients enrolled in the present study fulfilled the EULAR/ACR 2019 classification criteria for lupus [17]. The diagnosis of APS was based on the Sapporo (1999) or the Sydney criteria (2006); patients diagnosed with APS before 2006 were revised according to the Sydney criteria [18,19]. The study was approved by the Ethics Committee of our university (protocol number: 4879-2017) and was performed in agreement with the ethical standards of the Declaration of Helsinki.

### 2.2. Clinical and Laboratory Evaluation

All patients were routinely followed up throughout the studied period, and their medical records contained detailed information on medical history and treatments, as well as clinical symptoms, physical conditions, and laboratory and other findings of each visit. The following demographic and clinical data were analyzed: sex, age, age at diagnosis, disease duration, clinical symptoms and organ manifestations of lupus, comorbidities, laboratory results, immunoserological abnormalities and applied treatments during the disease course. The assessment of chronic organ damage was performed using the SLICC/ACR Damage Index (SDI) to identify chronic organ damage in lupus patients [20]. Routine diagnostic laboratory tests were performed at the Regional Immunology Laboratory of the Division of Clinical Immunology and at the Department of Laboratory Medicine, Faculty of Medicine, University of Debrecen. The clinical and laboratory data of patients were extracted from medical documentation and records for statistical analyses. The date of data collection was January 2020.

### 2.3. Statistical Analysis

The IBM SPSS version 22.0 software (SPSS Inc., Chicago, IL, USA) was used for statistical analysis. The Chi-square test and Fisher’s exact test were used to discriminate between patient groups, and Cramér’s V tests were used to measure the association between two nominal variables. Differences were considered statistically significant at *p* < 0.05.

## 3. Results

### 3.1. Main Analyses

The study population consisted of 554 Hungarian patients with SLE (496 women and 58 men) with a mean age ± standard deviation (SD) of 52.2 ± 14.46 years. Their mean age ± SD at the time of SLE diagnosis was 32.5 ± 12.55 years. We classified the lupus patients into three different groups: (1) APS patients, (2) APA positive patients not fulfilling APS clinical criteria, and (3) APA negative patients. A total of 113 patients (20.4%) were diagnosed with secondary APS; 247 patients (44.6%) belonged to the APA positive APS negative group, while 194 (35.0%) patients formed the APA negative group. All the patients were white adults, and the demographic characteristics of the patient groups did not differ significantly (Table 1).

### 3.2. The Prevalence of Antiphospholipid Antibodies

Among the antiphospholipid antibodies, anti-cardiolipin (IgG and/or IgM) occurred most frequently (58.48%), followed by anti-ß2-glycoprotein-I (IgG, IgM and/or IgA) (45.85%), and lupus anticoagulants (19.49%). Among the SLE patients with secondary APS, 23 patients (20.35%) were positive for one of the aforementioned markers, 44 (38.94%) had double positivity, and 46 patients (40.71%) were positive for all the three laboratory markers; therefore, nearly 80% of APS patients were double or triple positive. Regarding the APA positive group of lupus patients, 37.5% of them were single positive, 48.39% of them were double positive, and 14.11% of them were triple positive.

### 3.3. The Prevalence of Clinical Manifestations

As expected, the prevalence of thrombotic manifestations was significantly higher in patients with secondary APS compared with APA negative lupus patients. The prevalence of obstetric complications was also significantly higher in the group of SLE patients with secondary APS; however, it was relatively high also in the group of APA negative lupus patients. Table 2 shows the prevalences of these complications. We observed no significant difference between the three patient groups regarding other, non-thrombotic, inflammatory organ lesions usually common in SLE. Only central nervous system complications were more common in the secondary APS patients, but this difference was not significant compared with the other groups.

### 3.4. Anticoagulant and Antiplatelet Therapies

Table 3 shows the anticoagulant and antiplatelet therapies in the groups of APA positive lupus patients without APS and secondary APS patients. Low dose aspirin (43.72%) or clopidogrel (4.45%) were used as primary prevention in the APA positive lupus group. In this group, anticoagulants were given for other indications, such as atrial fibrillation. In the APS group, the largest proportion of patients received vitamin K antagonist therapy. Furthermore, 10.61% of APS patients receiving DOAC therapy were low-risk patients or had bleeding complications.

### 3.5. Differences between Patients of the APS Group Diagnosed with SLE before and since 2004

As a next step, we formed additional patient groups based on the year of diagnosis in order to compare the disease course of SLE patients diagnosed before and since 2004. For the sake of comparability, we collected the data of patients diagnosed before 2004 until January 2004; therefore, their mean follow-up time was 10.9 years. The patients diagnosed since 2004 were followed-up until January 2020 for an average of 9.9 years.

Table 4 demonstrates the observed differences among secondary APS patients. Acute myocardial infarction did not occur at all in the patients diagnosed since 2004, while it had developed in 10 out of 76 patients diagnosed with lupus before 2004. The prevalence of Raynaud-syndrome, central nervous system manifestations, polyarthritis and pericarditis significantly decreased in patients diagnosed since 2004. On the other hand, the prevalence of deep vein thrombosis and anemia, as well as the frequency of lupus anticoagulant, significantly increased in these patients.

### 3.6. Differences between Patients of the APA Positive non-APS Group Diagnosed with SLE before and since 2004

Table 5 shows the observed differences. We observed significantly decreased prevalence of central nervous system symptoms, psychiatric manifestations, discoid lupus and chronic kidney disease in patients diagnosed since 2004. The ratio of patients showing damage by SDI score also decreased significantly. However, hematologic pathologies such as leukopenia, anemia and thrombocytopenia became more common phenomena in this patient group.

### 3.7. Differences between Patients of APA Negative Group Diagnosed with SLE before and since 2004

Table 6 shows the observed differences. Most changes were similar to those observed in the APA positive group. Central nervous system manifestations, discoid lupus and pericarditis became less common, while the development of hematological manifestations, including leukopenia, anemia and thrombocytopenia became more frequent in the patients of the APA negative group diagnosed with lupus since 2004. The proportion of patients showing damage by SDI score also decreased significantly, although the prevalence of mucous ulcers significantly increased.

### 3.8. Comparison of the Medication and Disease Course of SLE Patients Diagnosed before and since 2004

The use of immunosuppressant drugs also shows significant changes. The administration of anti-malarial drugs (44.9% vs. 62%; *p* < 0.0001), mycophenolate mofetil (5.4% vs. 21.5%; *p* < 0.0001) and rituximab (1.7% vs. 6%; *p* = 0.049) significantly increased in SLE patients diagnosed since 2004, while there were no significant changes in the remaining immunosuppressive and immunomodulatory drugs.

During the investigated follow-up period, 21 new thrombotic incidents occurred (14 among patients before 2004, while 7 among patients diagnosed since 2004), from which 19 developed in the APS group, and two in the APA positive group. Among the latter, one developed deep vein thrombosis, while the other developed catastrophic antiphospholipid syndrome (CAPS), so they were transferred to the APS group, leaving 247 patients in the APA positive group out of the original 249. We examined the laboratory profile of the patients suffering from repeated thrombotic manifestations and revealed that all but two patients were double or triple positive for the elements of the APS laboratory criteria, namely lupus anticoagulant, anti-cardiolipin (aCL) and anti-beta2 glycoprotein I antibodies. Severe complications, such as HELLP syndrome or catastrophic antiphospholipid syndrome, appeared in triple positive patients only.

### 3.9. Comparison of the Mortality and Cause of Death

Mortality in the APS group was significantly higher compared with the other two groups. During the investigated follow-up period, 27 (23.7%) patients died in the APS group, from whom 23 (30.3%) patients were diagnosed before 2004 and 4 (10.8%) patients after 2004. There was no difference in the overall mortality rate between the APA positive and the APA negative groups (9.7% vs. 8.8%); however, when comparing the mortality rate based on the date of diagnosis, a strong improvement was observed in the subgroups of patients diagnosed after 2004, compared with those diagnosed before 2004 (APA positive: 3.6% vs. 12.9%; APA negative: 0.0% vs. 14.8%). Regarding the causes of death, infections, cardiovascular events and tumors were the leading causes in all three groups. The most common cause was infection (33.0%) in the APS group, followed by cardiovascular mortality (29.6%) and tumors (18.5%). In the APA positive group, the leading causes were cardiovascular events (33.3%), tumors (29%) and infections (25%), while in the APA negative group, the order of prevalence was tumors (41.0%), cardiovascular events (29.0%) and infections (23.5%).

## 4. Discussion

It is well established that the co-occurrence of systemic lupus erythematosus and antiphospholipid syndrome is a common phenomenon. There are numerous overlaps between the two diseases at both the laboratory and clinical levels, and the development of secondary APS increases the number of arterial and venous thromboembolic events in patients with SLE who are already at higher cardiovascular risk. Among our SLE patients, 20.4% suffered from secondary APS, 44.6% were APA positive without fulfilling the criteria of APS, while 35% did not have antiphospholipid antibodies. Of note, these ratios significantly differ from the results of a Columbian cross-sectional study [21], in which the ratio of the three groups appeared as follows: APS 9.3%, APA positive 30.8%, and APA negative 59.8%. However, the characteristics of SLE may differ between different ethnicities, and our ratios are similar to the observations based on the Spanish multicenter, hospital-based, retrospective, SLE registry (RELESSER-T) [7,22,23]. Among the 2398 European patients from the RELESSER-T registry, 1026 (42.8%) were classified into the SLE group, 555 (23.1%) into the SLE-APS group and 817 (34.1%) into the APA positive SLE group [7]. Therefore, our results are in line with the observations of the largest European registry of patients with SLE.

Beside thrombotic events, obstetric pathologies are the main clinical characteristic of APS; however, we also observed a high prevalence of obstetric complications in the group of APA negative lupus patients. The high number of unsuccessful pregnancies in lupus patients may be explained by a number of non-APS-related mechanisms, including complex immunopathological processes, underlying genetic factors, or side effects of previous treatments. Several immune mechanisms play an important role in a normal pregnancy, but in SLE, these immune mediated processes may change, and the consequential disturbed immune balance can contribute to pregnancy complications. These immunological changes include the loss of immune tolerance to the fetus, decreased absolute numbers of lymphocyte subpopulations, reduced numbers of B and NK cells, dysregulation of neutrophils and B cells, disrupted equilibrium of Th17 and Treg cells, and dysregulation of the TGF-β1/Treg cell axis [24]. Regarding other factors, the aberrant expression and the potential regulatory function of long noncoding RNAs in the placenta may play roles in the pathomechanism of lupus pregnancies [25]. Furthermore, three predictive gene biomarkers of adverse pregnancy outcomes were identified in pregnant women with SLE (SEZ6, NRAD1, and LPAR4) [26]. Based on these studies, the causes of pregnancy complications in lupus are very diverse and not fully known yet; therefore, further research is needed. 

In the present study, we did not find any significant difference in the non-APS-related symptoms and other organ manifestations of SLE between the three groups, contrary to the RELESSER-T register’s data. Ilgen et al. also found that symptoms specific to SLE, such as neurological symptoms, pleuritis, arthritis, nucleolar ANA positivity and endocarditis, were more common in the SLE-APS and SLE-APA positive groups than in the APA negative SLE population. Our own data do not confirm these findings [27].

Most of the patients in the SLE-APS group received anticoagulant therapy permanently, which follows the international protocols; moreover, near one fifth of the patients received both anticoagulants and antiplatelet therapy. Despite these measures, repeated thrombotic manifestations appeared in 16.8% of the patients. According to the data of the PROMISSE study, 44% of LA-positive APS women suffered pregnancy or obstetric complications even while taking the conservative prophylactic treatment [28]. Saraiva et al. examined an APS population and found that 38.4% of the patients included in their study developed recurrent thrombosis, of whom 40% were continuously anticoagulated [29]. The question is whether the use of anticoagulants and antiplatelet agents is sufficient in these cases, or whether other immunosuppressive treatment is also justified. Lifelong use of anticoagulants and antiplatelet agents is currently the recommended treatment for APS; however, several studies are ongoing regarding the use of immunosuppressive drugs in refractory cases. Immunomodulators and immunosuppressants can be considered, e.g., hydroxychloroquine, adalimumab, belimumab, rituximab, certolizumab, eculizumab, but currently, there are mainly only case reports available on their effectiveness [13]. There is increasing evidence that antimalarial drugs have a positive effect on antiphospholipid antibodies and also a beneficial effect on atherosclerotic processes, as well as reducing chronic organ damage [30].

Antiphospholipid antibodies play a key role in the development of APS; nevertheless, the data in the literature differ regarding the occurrence of individual antiphospholipid antibodies. The majority of the patients developing recurrent thrombotic events or severe APS complications, such as CAPS or HELLP (hemolysis, elevated liver enzymes and low platelets) syndrome showed double or triple antiphospholipid antibody positivity, indicating that multiple antibody positivity increases not only the thrombosis risk, but also the risk of recurrent thrombosis. This observation is consistent with the data of other studies, which reported that multiple positivity results in a higher risk of thrombosis, primarily deep vein thrombosis and pulmonary embolism [7,22].

Considering the improving diagnostic protocols that lead to earlier diagnoses, the more differentiated novel immunosuppressive therapies and the useful international guidelines, the clinical course of SLE and APS has potentially changed substantially in the past two decades. In our study, we focused on the assessment of these changes in the disease course of lupus, with a special emphasis on secondary APS and APA positivity. Among the thrombotic events, we observed an increase in the prevalence of deep vein thrombosis in the last decades, which could be caused by the fact that more attention is paid to the possibly underlying APS and that the diagnostic procedure of deep vein thrombosis has become more accurate. Acute myocardial infarct did not occur in SLE patients diagnosed in the past 15 years, which could be related to the use of low-dose aspirin as primary prevention and the significant increase in the use of chloroquine, which display beneficial effects on atherosclerosis [31,32]. We did not observe any changes regarding the further thrombotic or non-APS-criteria symptoms. Based on our results, since 2004, hematological complications such as leukopenia, anemia and thrombocytopenia increased significantly in the APA positive and APA negative patient groups, but not in the APS group; therefore, these are thought to be manifestations of lupus rather than the consequences of APS. Regarding our total SLE population, the prevalence of both central nervous system involvement and chronic organ damage have significantly decreased in the past decades, although the highest rates of central nervous system involvement and organ damage developed in the APS group. New thrombotic events occurred in the APS group in spite of the use of anticoagulants and thrombocyte aggregation inhibitors. Nineteen (16.8%) of our SLE-APS patients developed new thrombotic manifestations. The number of new thrombotic events among patients diagnosed before 2004 is twice as high as the number of cases developed in patients diagnosed after 2004. This suggests that the course of SLE-APS did indeed improve, despite the fact that the mortality rate was significantly higher in this patient group. The causes of death were consistent with the data of other centers where cardiovascular morbidities, infections and tumors were the most common causes [10,33].

## 5. Conclusions

Close monitoring of SLE patients for thrombotic events and administration of immunomodulant or immunosuppressive treatment alongside anticoagulation treatment of the SLE-APS patients should be of high priority, since the co-occurrence of APS with SLE is a risk factor in the formation of new thrombotic events, in spite of the fine-tuned use of anticoagulants and thrombocyte aggregation inhibitors. Based on our observations, in the last two decades, the prevalence of neurological manifestations and acute myocardial infarction decreased significantly. Moreover, the degree of chronic organ damage became more favorable in all three patient groups; however, the number of patients developing chronic organ damage remained high in the APS group. Overall, the course of SLE and APS has become more favorable, although a prospective, multi-center study is needed to confirm our finding.

## Figures and Tables

**Table 1:** Demographic characteristics of the subgroups of SLE patients.

Characteristics	Total (*n* = 554)	APA− (*n* = 194)	APA+ (*n* = 247)	APS (*n* = 113)	*p* Value
Age, years	52.2 ± 14.46	51.1 ± 15.30	52.6 ± 14.39	54.9 ± 14.49	n.s.
Age at diagnosis, years	32.5 ± 12.55	32.7 ± 12.68	32.4 ± 12.55	34.5 ± 11.79	n.s.
Duration of disease, years	18.3 ± 10.73	17.0 ± 11.79	19.1 ± 10.10	18.5 ± 10.44	n.s.
Sex, Female/Male	496/58	172/22	226/21	98/15	n.s.

Data are displayed as mean ± standard deviation. APA−, antiphospholipid antibody negative; APA+, antiphospholipid antibody positive without APS; APS, antiphospholipid syndrome; n.s., non-significant.

**Table 2:** Prevalence of thrombotic manifestations and pregnancy complications in the groups of APA negative lupus patients and secondary APS patients.

Complications	APA− (*n* = 194)	APS (*n* = 113)	*p* Value
AMI	1.03%	8.85%	*p* = 0.002
Stroke	2.58%	30.09%	*p* < 0.0001
DVT	4.12%	61.95%	*p* < 0.0001
Pulmonary embolism	1.55%	21.24%	*p* < 0.0001
Obstetric complications	8.25%	24.78%	*p* = 0.001

Prevalences are given in percentages. APA−, antiphospholipid antibody negative; APS, antiphospholipid syndrome; AMI, acute myocardial infarction; DVT, deep vein thrombosis.

**Table 3:** Administration of anticoagulant and antiplatelet therapies in the groups of APA positive lupus patients without APS and secondary APS patients.

Treatment	APA+ (*n* = 247)	APS (*n* = 113)	*p* Value
Vitamin K antagonists	4.04%	55.75%	*p* < 0.0001
LMWHs	1.61%	8.85%	*p* = 0.001
ASA/Clopidogrel	48.17%	45.13%	n.s.
Anticoagulant + TAIs	2.83%	19.46%	*p* < 0.0001
DOACs	0.8%	10.61%	*p* < 0.0001

Prevalences are given in percentages. APA+, antiphospholipid antibody positive without APS; APS, antiphospholipid syndrome; LMWHs, low-molecular-weight heparins; ASA, acetylsalicylic acid; TAIs, thrombocyte aggregation inhibitors; DOACs, direct oral anticoagulants; n.s., non-significant.

**Table 4:** Differences in the clinical symptoms and laboratory findings between patients of the APS group diagnosed with SLE before and since 2004.

Complications	Before 2004 (*n* = 76)	Since 2004 (*n* = 37)	*p* Value
AMI	13.2%	0.00%	*p* = 0.021
DVT	56.6%	75.7%	*p* = 0.049
CNS	52.6%	27.0%	*p* = 0.010
Raynaud syndrome	48.7%	27.0%	*p* = 0.024
Polyarthritis	89.5%	67.6%	*p* = 0.004
Pericarditis	22.4%	0.00%	*p* = 0.002
Lupus anticoagulant	42.1%	62.2%	*p* = 0.045
Anemia	64.5%	83.8%	*p* = 0.034

Prevalences are given in percentages. APS, antiphospholipid syndrome; AMI, acute myocardial infarction; DVT, deep vein thrombosis; CNS, central nervous system.

**Table 5:** Differences in the clinical symptoms and laboratory findings between patients of the APA positive non-APS group diagnosed with SLE before and since 2004.

Complications	Before 2004 (*n* = 163)	Since 2004 (*n* = 84)	*p* Value
CNS	29.4%	16.7%	*p* = 0.028
Psychiatric disease	28.2%	11.9%	*p* = 0.004
Discoid lupus	17.8%	8.3%	*p* = 0.046
Anti-CL antibody	92.6%	83.3%	*p* = 0.024
Anemia	60.7%	85.7%	*p* < 0.0001
Leukopenia	65.6%	78.6%	*p* = 0.036
Thrombocytopenia	27.6%	46.4%	*p* = 0.003
CKD	24.5%	9.5%	*p* = 0.005
SDI ≥ 1	71.8%	29.8%	*p* < 0.0001

Prevalences are given in percentages. APA+, antiphospholipid antibody positive without APS; APS, antiphospholipid syndrome; CNS, central nervous system; CL, cardiolipin; CKD, chronic kidney disease; SDI, SLICC/ACR Damage Index.

**Table 6:** Differences in the clinical symptoms and laboratory findings between patients of the APA negative group diagnosed with SLE before and since 2004.

Complications	Before 2004 (*n* = 115)	Since 2004 (*n* = 79)	*p* Value
CNS	20.8%	8.86%	*p* = 0.025
Discoid lupus	23.5%	11.4%	*p* = 0.033
Mucous ulcers	5.2%	15.2%	*p* = 0.019
Pericarditis	27.0%	7.6%	*p* = 0.001
Anemia	36.5%	82.3%	*p* < 0.0001
Leukopenia	46.1%	76.0%	*p* < 0.0001
Thrombocytopenia	15.7%	38.0%	*p* = 0.0004
SDI ≥ 1	66.0%	19.0%	*p* < 0.0001

Prevalences are given in percentages. APA−, antiphospholipid antibody negative; CNS, central nervous system; SDI, SLICC/ACR Damage Index.

## Data Availability

We cannot provide public access to individual data due to participant privacy stipulations in accordance with ethical guidelines. Upon reasonable request, qualifying researchers may apply to access an aggregated dataset by contacting the corresponding author.
